# Tris(*O*-cyclo­hexyl dithio­carbonato-κ*S*)anti­mony(III)

**DOI:** 10.1107/S1600536808040804

**Published:** 2008-12-10

**Authors:** Wenkuan Li, Handong Yin, Liyuan Wen, Daqi Wang

**Affiliations:** a College of Chemistry and Chemical Engineering, Liaocheng University, Shandong 252059, People’s Republic of China

## Abstract

In the mol­ecule of the title compound, [Sb(C_7_H_11_OS_2_)_3_], the anti­mony(III) is coordinated by the S atoms of three *O*-alkyl xanthate groups acting as monodentate ligands, forming a distorted trigonal-pyramidal coordination.

## Related literature

For the biological activity of anti­mony(III) complexes, see: Tiekink (2002 ▶); Wang *et al.* (2005 ▶). For a related structure, see: Baba *et al.* (2001 ▶).
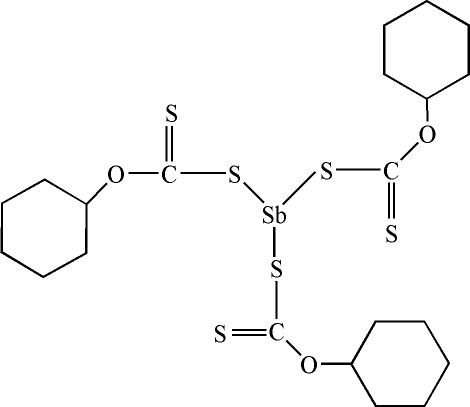


## Experimental

### 

#### Crystal data

[Sb(C_7_H_11_OS_2_)_3_]
*M* *_r_* = 646.58
Monoclinic, 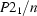

*a* = 9.4187 (12) Å
*b* = 18.866 (2) Å
*c* = 15.8583 (18) Å
β = 93.944 (2)°
*V* = 2811.2 (6) Å^3^
*Z* = 4
Mo *K*α radiation
μ = 1.45 mm^−1^
*T* = 298 (2) K
0.30 × 0.25 × 0.18 mm

#### Data collection

Bruker SMART CCD area-detector diffractometer
Absorption correction: multi-scan (*SADABS*; Sheldrick, 1996 ▶) *T*_min_ = 0.664, *T*_max_ = 0.773
14030 measured reflections
4946 independent reflections
3183 reflections with *I* > 2σ(*I*)
*R*_int_ = 0.071

#### Refinement

*R*[*F* ^2^ > 2σ(*F* ^2^)] = 0.047
*wR*(*F* ^2^) = 0.127
*S* = 1.00
4946 reflections
280 parameters
90 restraints
H-atom parameters constrained
Δρ_max_ = 0.86 e Å^−3^
Δρ_min_ = −0.60 e Å^−3^

### 

Data collection: *SMART* (Siemens, 1996 ▶); cell refinement: *SAINT* (Siemens, 1996 ▶); data reduction: *SAINT*; program(s) used to solve structure: *SHELXS97* (Sheldrick, 2008 ▶); program(s) used to refine structure: *SHELXL97* (Sheldrick, 2008 ▶); molecular graphics: *SHELXTL* (Sheldrick, 2008 ▶); software used to prepare material for publication: *SHELXTL*.

## Supplementary Material

Crystal structure: contains datablocks I, global. DOI: 10.1107/S1600536808040804/rz2271sup1.cif

Structure factors: contains datablocks I. DOI: 10.1107/S1600536808040804/rz2271Isup2.hkl

Additional supplementary materials:  crystallographic information; 3D view; checkCIF report

## Figures and Tables

**Table 1:** Selected bond lengths (Å)

Sb1—S5	2.5072 (14)
Sb1—S1	2.5123 (17)
Sb1—S3	2.5140 (15)

**Fig. 1.**
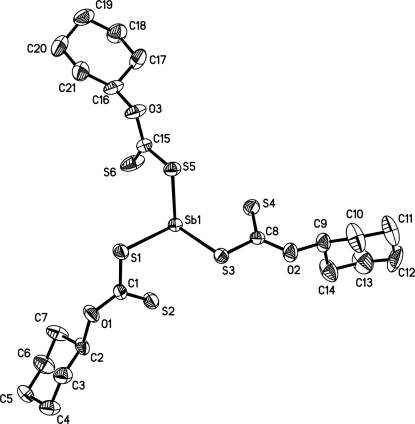
The molecular structure of the title compound, showing 50% probability displacement ellipsoids. H atoms are omitted for clarity.

**Fig. 2.**
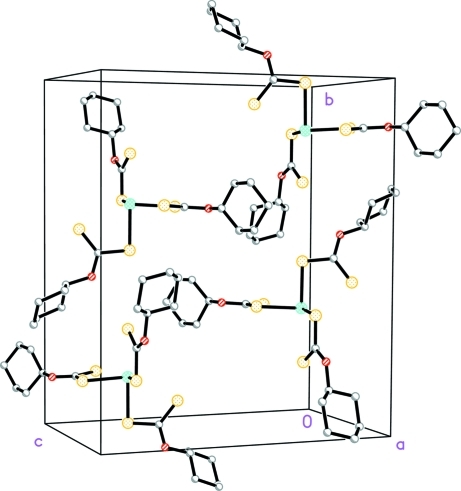
Packing diagram of the title compound viewed approximately along the a axis. H atoms are omitted for clarity.

## supplementary crystallographic information

### Comment

The coordination chemistry of antimony has both a practical and theoretical interest.The medicinal and cosmetic use of antimony complexes goes back at least to the Egyptians. Potassium antimony tartrate or tartar emetic was widely used until the early 1900s despite the somewhat toxic nature of the material. On the other hand, antimony complexes have been reported with good cytotoxicity and antitumor activities (Tiekink, 2002; Wang *et al.*, 2005). As a part of our ongoing investigations in the field we have synthesized the title compound and determined its crystal structure.

In the title compound, the antomony(III) ion lies on a *pseudo* threefold axis (Fig. 1). The *O*-alkylxanthate ligands coordinate to the antimony(III) ion in a monodentate mode, with Sb—S distances ranging from 2.5072 (14) to 2.5140 (15) Å (Table 1), to form a trigonal pyramidal geometry. The separations between the Sb atom and the S atoms (S2, S4, S6) not involved in the coordination range from 2.9458 (16) to 3.0617 (17) Å. By taking into account these atoms, the coordination geometry may be described alternatively as distorted octahedral, in which the lone electron pair of the Sb atom projects out of the triangular face defined by the S2, S4 and S6 atoms, thereby elongating these bonds. The crystal packing (Fig. 2) is stabilized mainly by van der Waals interactions. The crystal structure of a similar compound ([Sb(C_8_H_14_NS_2_)_3_]) have been reported recently (Baba *et al.*, 2001).

### Experimental

The title compoud were prepared by reaction of antimony(III) chloride (0.114 g,0.5 mmol) with the corresponding sodium *O*-alkylxanthate (0.2974 g,1.5 mmol) in absolute benzene. After stirring for 7 h at room temperature, the yellow paste obtained ws filtered. Yellow crystals suitable for X-ray analysis were obtained by slow evaporation of a n-hexane/dichloromethane (1:2 v/v) solution over a period of ten days (yield 90%; m.p. 450 K). Anal. Calcd (%) for C_21_H_33_O_3_S_6_Sb (Mr = 646.65): C, 39.00; H, 5.14; O, 7.42; S, 29.75; Sb, 18.82 Found (%): C, 39.02; H, 5.10; O, 7.40; S, 29.77; Sb, 18.79

### Refinement

All H atoms were positioned geometrically (C—H = 0.97–0.98 Å) and refined as riding with *U*_iso_(H) = 1.2 *U*_eq_(C).

### Crystal data

**Table:**

[Sb(C_7_H_11_OS_2_)_3_]	*F*(000) = 1316
*M**_r_* = 646.58	*D*_x_ = 1.528 Mg m^−^^3^
Monoclinic, *P*2_1_/*n*	Mo *K*α radiation, λ = 0.71073 Å
Hall symbol: -P 2yn	Cell parameters from 3706 reflections
*a* = 9.4187 (12) Å	θ = 2.5–25.0°
*b* = 18.866 (2) Å	µ = 1.45 mm^−^^1^
*c* = 15.8583 (18) Å	*T* = 298 K
β = 93.944 (2)°	Block, yellow
*V* = 2811.2 (6) Å^3^	0.30 × 0.25 × 0.18 mm
*Z* = 4	

### Data collection

**Table:**

Bruker SMART CCD area-detector diffractometer	4946 independent reflections
Radiation source: fine-focus sealed tube	3183 reflections with *I* > 2σ(*I*)
graphite	*R*_int_ = 0.071
φ and ω scans	θ_max_ = 25.0°, θ_min_ = 1.7°
Absorption correction: multi-scan (*SADABS*; Sheldrick, 1996)	*h* = −11→11
*T*_min_ = 0.664, *T*_max_ = 0.773	*k* = −15→22
14030 measured reflections	*l* = −16→18

### Refinement

**Table:**

Refinement on *F*^2^	Primary atom site location: structure-invariant direct methods
Least-squares matrix: full	Secondary atom site location: difference Fourier map
*R*[*F*^2^ > 2σ(*F*^2^)] = 0.047	Hydrogen site location: inferred from neighbouring sites
*wR*(*F*^2^) = 0.127	H-atom parameters constrained
*S* = 1.00	*w* = 1/[σ^2^(*F*_o_^2^) + (0.0583*P*)^2^] where *P* = (*F*_o_^2^ + 2*F*_c_^2^)/3
4946 reflections	(Δ/σ)_max_ < 0.001
280 parameters	Δρ_max_ = 0.86 e Å^−^^3^
90 restraints	Δρ_min_ = −0.60 e Å^−^^3^

### Fractional atomic coordinates and isotropic or equivalent isotropic displacement parameters (Å^2^)

**Table:**

	*x*	*y*	*z*	*U*_iso_*/*U*_eq_	
Sb1	0.23869 (4)	0.14457 (2)	0.75101 (2)	0.04805 (16)	
O1	0.2841 (5)	−0.0631 (2)	0.6250 (2)	0.0678 (12)	
O2	0.6257 (4)	0.2720 (2)	0.7766 (3)	0.0686 (11)	
O3	−0.0197 (4)	0.1350 (3)	0.9707 (2)	0.0710 (13)	
S1	0.25550 (19)	0.01168 (9)	0.75366 (9)	0.0625 (5)	
S2	0.2947 (2)	0.07274 (9)	0.58338 (10)	0.0703 (5)	
S3	0.50432 (16)	0.15233 (7)	0.77844 (10)	0.0522 (4)	
S4	0.34846 (18)	0.28996 (8)	0.77444 (12)	0.0685 (5)	
S5	0.21960 (15)	0.13712 (9)	0.90767 (9)	0.0551 (4)	
S6	−0.05683 (19)	0.14457 (13)	0.80641 (10)	0.0940 (7)	
C1	0.2778 (6)	0.0043 (3)	0.6466 (3)	0.0556 (15)	
C2	0.2862 (7)	−0.0849 (3)	0.5356 (3)	0.0608 (17)	
H2	0.3328	−0.0487	0.5028	0.073*	
C3	0.3659 (7)	−0.1531 (4)	0.5347 (4)	0.0683 (19)	
H3A	0.3241	−0.1869	0.5718	0.082*	
H3B	0.4641	−0.1454	0.5552	0.082*	
C4	0.3606 (7)	−0.1822 (4)	0.4458 (4)	0.075 (2)	
H4A	0.4093	−0.1499	0.4099	0.090*	
H4B	0.4096	−0.2274	0.4459	0.090*	
C5	0.2105 (7)	−0.1918 (3)	0.4107 (4)	0.0684 (18)	
H5A	0.2105	−0.2078	0.3526	0.082*	
H5B	0.1653	−0.2281	0.4429	0.082*	
C6	0.1273 (8)	−0.1250 (4)	0.4138 (4)	0.088 (2)	
H6A	0.0287	−0.1343	0.3952	0.106*	
H6B	0.1644	−0.0906	0.3755	0.106*	
C7	0.1347 (8)	−0.0944 (4)	0.5030 (4)	0.079 (2)	
H7A	0.0861	−0.0491	0.5025	0.095*	
H7B	0.0870	−0.1261	0.5400	0.095*	
C8	0.4972 (6)	0.2437 (3)	0.7757 (3)	0.0487 (14)	
C9	0.6450 (7)	0.3490 (3)	0.7713 (4)	0.0678 (17)	
H9	0.5588	0.3726	0.7884	0.081*	
C10	0.7653 (11)	0.3685 (4)	0.8306 (6)	0.119 (3)	
H10A	0.8486	0.3410	0.8187	0.143*	
H10B	0.7422	0.3583	0.8881	0.143*	
C11	0.7964 (12)	0.4479 (4)	0.8213 (6)	0.129 (3)	
H11A	0.7172	0.4750	0.8404	0.155*	
H11B	0.8805	0.4600	0.8571	0.155*	
C12	0.8189 (10)	0.4673 (5)	0.7347 (7)	0.129 (4)	
H12	0.9001	0.4896	0.7174	0.154*	
C13	0.6975 (12)	0.4471 (4)	0.6787 (5)	0.123 (3)	
H13A	0.7161	0.4591	0.6210	0.148*	
H13B	0.6147	0.4737	0.6935	0.148*	
C14	0.6660 (11)	0.3674 (4)	0.6843 (5)	0.102 (2)	
H14A	0.5813	0.3559	0.6487	0.123*	
H14B	0.7449	0.3404	0.6645	0.123*	
C15	0.0345 (6)	0.1386 (3)	0.8972 (3)	0.0528 (15)	
C16	−0.1749 (6)	0.1372 (4)	0.9774 (4)	0.0660 (19)	
H16	−0.2231	0.1328	0.9209	0.079*	
C17	−0.2112 (7)	0.2063 (4)	1.0141 (5)	0.081 (2)	
H17A	−0.1850	0.2441	0.9767	0.097*	
H17B	−0.1576	0.2125	1.0680	0.097*	
C18	−0.3717 (7)	0.2105 (4)	1.0267 (5)	0.094 (2)	
H18A	−0.3920	0.2539	1.0563	0.112*	
H18B	−0.4248	0.2114	0.9721	0.112*	
C19	−0.4176 (7)	0.1482 (5)	1.0765 (5)	0.088 (2)	
H19A	−0.3746	0.1513	1.1338	0.106*	
H19B	−0.5201	0.1496	1.0794	0.106*	
C20	−0.3769 (8)	0.0802 (4)	1.0387 (5)	0.086 (2)	
H20A	−0.4261	0.0752	0.9832	0.103*	
H20B	−0.4062	0.0413	1.0737	0.103*	
C21	−0.2153 (7)	0.0760 (4)	1.0305 (4)	0.080 (2)	
H21A	−0.1651	0.0785	1.0859	0.096*	
H21B	−0.1909	0.0317	1.0041	0.096*	

### Atomic displacement parameters (Å^2^)

**Table:**

	*U*^11^	*U*^22^	*U*^33^	*U*^12^	*U*^13^	*U*^23^
Sb1	0.0516 (3)	0.0496 (3)	0.0438 (2)	−0.00345 (19)	0.00902 (16)	−0.0031 (2)
O1	0.111 (4)	0.047 (3)	0.047 (2)	−0.013 (2)	0.014 (2)	−0.010 (2)
O2	0.061 (3)	0.042 (2)	0.103 (3)	−0.003 (2)	0.007 (2)	0.001 (2)
O3	0.050 (3)	0.125 (4)	0.040 (2)	0.001 (3)	0.0127 (18)	0.003 (2)
S1	0.0940 (13)	0.0493 (10)	0.0461 (8)	−0.0114 (9)	0.0185 (8)	−0.0035 (8)
S2	0.1089 (14)	0.0522 (11)	0.0518 (9)	−0.0140 (10)	0.0208 (9)	−0.0035 (8)
S3	0.0502 (9)	0.0390 (8)	0.0681 (9)	0.0022 (7)	0.0102 (7)	−0.0032 (7)
S4	0.0612 (10)	0.0473 (10)	0.0995 (13)	0.0073 (8)	0.0244 (9)	−0.0028 (9)
S5	0.0482 (9)	0.0748 (11)	0.0425 (7)	−0.0002 (8)	0.0054 (6)	−0.0058 (8)
S6	0.0518 (10)	0.185 (2)	0.0455 (9)	0.0012 (12)	0.0042 (7)	0.0083 (12)
C1	0.067 (4)	0.046 (4)	0.054 (4)	−0.013 (3)	0.010 (3)	−0.009 (3)
C2	0.095 (5)	0.051 (4)	0.036 (3)	−0.019 (4)	0.005 (3)	−0.008 (3)
C3	0.054 (4)	0.086 (5)	0.064 (4)	0.001 (4)	0.001 (3)	−0.012 (4)
C4	0.070 (5)	0.095 (6)	0.062 (4)	0.011 (4)	0.012 (3)	−0.019 (4)
C5	0.085 (5)	0.054 (4)	0.065 (4)	0.001 (4)	−0.001 (3)	−0.019 (3)
C6	0.097 (6)	0.092 (6)	0.072 (5)	0.019 (5)	−0.020 (4)	−0.021 (4)
C7	0.085 (5)	0.085 (6)	0.067 (4)	0.038 (4)	−0.005 (4)	−0.021 (4)
C8	0.054 (4)	0.047 (4)	0.046 (3)	0.000 (3)	0.013 (3)	−0.004 (3)
C9	0.072 (4)	0.041 (4)	0.091 (4)	−0.009 (3)	0.007 (3)	0.005 (3)
C10	0.163 (7)	0.068 (5)	0.118 (6)	−0.025 (5)	−0.045 (5)	0.002 (5)
C11	0.161 (7)	0.068 (5)	0.149 (6)	−0.031 (5)	−0.052 (6)	−0.014 (5)
C12	0.090 (7)	0.087 (7)	0.213 (11)	−0.048 (6)	0.039 (7)	0.014 (7)
C13	0.186 (7)	0.078 (5)	0.108 (5)	−0.025 (5)	0.020 (5)	0.021 (5)
C14	0.166 (7)	0.053 (4)	0.087 (5)	−0.024 (5)	0.003 (5)	0.002 (4)
C15	0.050 (3)	0.058 (4)	0.052 (3)	−0.002 (3)	0.015 (3)	−0.003 (3)
C16	0.045 (4)	0.112 (6)	0.042 (3)	0.000 (4)	0.010 (3)	0.003 (4)
C17	0.063 (5)	0.066 (5)	0.116 (6)	−0.002 (4)	0.024 (4)	0.023 (5)
C18	0.061 (5)	0.101 (7)	0.122 (6)	0.010 (4)	0.023 (4)	−0.001 (5)
C19	0.054 (4)	0.139 (8)	0.073 (5)	−0.015 (5)	0.018 (4)	−0.008 (5)
C20	0.072 (5)	0.106 (7)	0.079 (5)	−0.034 (5)	0.002 (4)	0.020 (5)
C21	0.071 (5)	0.075 (5)	0.096 (5)	−0.012 (4)	0.015 (4)	−0.005 (4)

### Geometric parameters (Å, °)

**Table:**

Sb1—S5	2.5072 (14)	C9—C10	1.469 (10)
Sb1—S1	2.5123 (17)	C9—H9	0.9800
Sb1—S3	2.5140 (15)	C10—C11	1.535 (10)
O1—C1	1.320 (6)	C10—H10A	0.9700
O1—C2	1.477 (6)	C10—H10B	0.9700
O2—C8	1.322 (6)	C11—C12	1.451 (11)
O2—C9	1.468 (7)	C11—H11A	0.9700
O3—C15	1.306 (6)	C11—H11B	0.9700
O3—C16	1.474 (7)	C12—C13	1.449 (11)
S1—C1	1.731 (5)	C12—H12	0.9300
S2—C1	1.649 (6)	C13—C14	1.537 (10)
S3—C8	1.726 (6)	C13—H13A	0.9700
S4—C8	1.649 (6)	C13—H13B	0.9700
S5—C15	1.740 (6)	C14—H14A	0.9700
S6—C15	1.630 (6)	C14—H14B	0.9700
C2—C3	1.490 (8)	C16—C17	1.475 (9)
C2—C7	1.496 (9)	C16—C21	1.493 (9)
C2—H2	0.9800	C16—H16	0.9800
C3—C4	1.511 (8)	C17—C18	1.541 (9)
C3—H3A	0.9700	C17—H17A	0.9700
C3—H3B	0.9700	C17—H17B	0.9700
C4—C5	1.493 (8)	C18—C19	1.497 (10)
C4—H4A	0.9700	C18—H18A	0.9700
C4—H4B	0.9700	C18—H18B	0.9700
C5—C6	1.487 (9)	C19—C20	1.479 (10)
C5—H5A	0.9700	C19—H19A	0.9700
C5—H5B	0.9700	C19—H19B	0.9700
C6—C7	1.524 (8)	C20—C21	1.538 (9)
C6—H6A	0.9700	C20—H20A	0.9700
C6—H6B	0.9700	C20—H20B	0.9700
C7—H7A	0.9700	C21—H21A	0.9700
C7—H7B	0.9700	C21—H21B	0.9700
C9—C14	1.450 (10)		
			
S5—Sb1—S1	86.34 (5)	H10A—C10—H10B	108.3
S5—Sb1—S3	88.29 (5)	C12—C11—C10	112.3 (8)
S1—Sb1—S3	89.68 (5)	C12—C11—H11A	109.1
C1—O1—C2	121.3 (5)	C10—C11—H11A	109.1
C8—O2—C9	121.0 (5)	C12—C11—H11B	109.1
C15—O3—C16	120.9 (4)	C10—C11—H11B	109.1
C1—S1—Sb1	94.3 (2)	H11A—C11—H11B	107.9
C8—S3—Sb1	91.0 (2)	C13—C12—C11	110.7 (7)
C15—S5—Sb1	92.54 (18)	C13—C12—H12	124.7
O1—C1—S2	126.1 (4)	C11—C12—H12	124.7
O1—C1—S1	110.0 (4)	C12—C13—C14	111.7 (8)
S2—C1—S1	123.8 (3)	C12—C13—H13A	109.3
O1—C2—C3	106.8 (5)	C14—C13—H13A	109.3
O1—C2—C7	106.9 (5)	C12—C13—H13B	109.3
C3—C2—C7	111.2 (5)	C14—C13—H13B	109.3
O1—C2—H2	110.6	H13A—C13—H13B	107.9
C3—C2—H2	110.6	C9—C14—C13	109.2 (6)
C7—C2—H2	110.6	C9—C14—H14A	109.8
C2—C3—C4	109.8 (5)	C13—C14—H14A	109.8
C2—C3—H3A	109.7	C9—C14—H14B	109.8
C4—C3—H3A	109.7	C13—C14—H14B	109.8
C2—C3—H3B	109.7	H14A—C14—H14B	108.3
C4—C3—H3B	109.7	O3—C15—S6	125.2 (4)
H3A—C3—H3B	108.2	O3—C15—S5	111.4 (4)
C5—C4—C3	111.2 (5)	S6—C15—S5	123.4 (3)
C5—C4—H4A	109.4	O3—C16—C17	108.1 (5)
C3—C4—H4A	109.4	O3—C16—C21	108.2 (5)
C5—C4—H4B	109.4	C17—C16—C21	112.7 (5)
C3—C4—H4B	109.4	O3—C16—H16	109.3
H4A—C4—H4B	108.0	C17—C16—H16	109.3
C6—C5—C4	111.8 (6)	C21—C16—H16	109.3
C6—C5—H5A	109.2	C16—C17—C18	110.7 (6)
C4—C5—H5A	109.2	C16—C17—H17A	109.5
C6—C5—H5B	109.2	C18—C17—H17A	109.5
C4—C5—H5B	109.2	C16—C17—H17B	109.5
H5A—C5—H5B	107.9	C18—C17—H17B	109.5
C5—C6—C7	111.1 (5)	H17A—C17—H17B	108.1
C5—C6—H6A	109.4	C19—C18—C17	110.5 (6)
C7—C6—H6A	109.4	C19—C18—H18A	109.6
C5—C6—H6B	109.4	C17—C18—H18A	109.6
C7—C6—H6B	109.4	C19—C18—H18B	109.6
H6A—C6—H6B	108.0	C17—C18—H18B	109.6
C2—C7—C6	110.4 (6)	H18A—C18—H18B	108.1
C2—C7—H7A	109.6	C20—C19—C18	112.0 (6)
C6—C7—H7A	109.6	C20—C19—H19A	109.2
C2—C7—H7B	109.6	C18—C19—H19A	109.2
C6—C7—H7B	109.6	C20—C19—H19B	109.2
H7A—C7—H7B	108.1	C18—C19—H19B	109.2
O2—C8—S4	124.3 (4)	H19A—C19—H19B	107.9
O2—C8—S3	111.6 (4)	C19—C20—C21	111.3 (6)
S4—C8—S3	124.1 (3)	C19—C20—H20A	109.4
C14—C9—O2	108.5 (6)	C21—C20—H20A	109.4
C14—C9—C10	113.6 (7)	C19—C20—H20B	109.4
O2—C9—C10	107.6 (6)	C21—C20—H20B	109.4
C14—C9—H9	109.0	H20A—C20—H20B	108.0
O2—C9—H9	109.0	C16—C21—C20	107.5 (6)
C10—C9—H9	109.0	C16—C21—H21A	110.2
C9—C10—C11	109.0 (7)	C20—C21—H21A	110.2
C9—C10—H10A	109.9	C16—C21—H21B	110.2
C11—C10—H10A	109.9	C20—C21—H21B	110.2
C9—C10—H10B	109.9	H21A—C21—H21B	108.5
C11—C10—H10B	109.9		
